# Early historical report of alcohol hepatotoxicity in *Minooye Kherad*, a Pahlavi manuscript in Ancient Persia, 6^th^ century CE

**DOI:** 10.22088/cjim.13.2.431

**Published:** 2022

**Authors:** Hanan Hamdi, Samaneh Soleymani, Arman Zargaran

**Affiliations:** 1 Department of Traditional Pharmacy, School of Persian Medicine, Tehran University of Medical Sciences, Tehran, Iran; 2 School of Advanced Technologies in Medicine, Iran University of Medical Sciences, Tehran, Iran; 3 Department of History of Medicine, School of Persian Medicine, Tehran University of Medical Sciences, Tehran, Iran

**Keywords:** Alcoholism, History of medicine, Liver injury, Persian Medicine, Wine

## Abstract

**Background:**

Historical evidence revealed that alcoholic beverages have been produced, used and abused thousands of years before the discovery of alcohol by Rhazes for medical purposes. Alcohol-induced liver disease (e.g., steatosis, steatohepatitis, fibrosis and cirrhosis) is one of the most prevalent causes of chronic liver disease all over the world. This study aims to find the early report of this complication in an ancient Persian historical text.

**Methods:**

In this study, the book of *Minooye Kherad*, a Zoroastrian manuscript on wisdom which was written in the late Sassanid Empire (224–637 CE) is reviewed.

**Results:**

However, the concept of alcohol hepatotoxicity as one of the most important complications of alcoholism is a new terminology, by researching historical documents it can be found that one of the oldest reports of benefits and disadvantages of drinking wine focusing on liver complications is mentioned in the book of *Minooye*.

**Conclusion:**

Description of the liver disease and damage caused by excessive alcohol consumption in this valuable book can be considered as the early report of hepatotoxicity of alcoholic beverages in the medical history.

Although alcohol and its use for medical purposes was discovered and introduced by Rhazes (865-925 AD), a Persian pioneer physician, philosopher and alchemist (1), several kinds of alcoholic beverages have been produced, consumed and abused by humans for thousands of years (2). However, there are different opinions as to when humans first began to create or be familiar with alcoholic drinks, archaeological and historical evidence revealed that the fermentation of grains into beer such as grape juice into wine dated back about 20,000 years as an ancient custom (2).

 It appears that fermented mare's milk was one of the first alcoholic beverage in ancient Siberia that nowadays known as “Kumis” in some parts of Russia (2). Different jars have been discovered in excavations around the world that were applied for storing alcoholic beverages in numerous religious ceremonies, social gatherings including Northern China (7000–6600 BCE) (3), Persia (5400–5000 BCE) (4), ancient Egypt (4000 BCE), Babylonians (2700 BCE) (5), Mexico (1000 BCE) (4), Greece (700 BCE) (6). In the late 19^th^ century, the world observed the birth of a new disease concept of alcoholism by Magnus Huss, the Swedish physician in 1868 (7, 8). Alcoholism (alcohol addiction and abuse) is a complex disorder related to emotional, social, economic and biological factors, which often causes health complications including coronary artery disease, cardiomyopathy, central nervous system disorders and liver cirrhosis (9). 

Liver, the most affected organ is the primary site for alcohol metabolism. Excessive alcohol drinking results to fibrosis, scar tissue, cirrhosis and death of liver cells (3). The concept of alcohol hepatotoxicity is now widely accepted but association between alcohol abuse and liver disorders has been identified over the history of medicine (10). 

It has been reported that Simon Seth was one of physicians, in the 11^th^ century who spoke about the influence of excessive wine drinking on human health specifically that associated alcohol with the inflammation and damage of the liver (1, 7).

Although this term is a new concept in medical sciences, by researching historical documents, one of the oldest reports of the benefits and disadvantages of wine drinking especially focusing on liver effects in the "*Minooy Kherad*" can be found, a book written during the Sasanian era (224–637 CE) in Iran. This study attempted to introduce these properties. 

**Sassanid Era (224–637 CE): **The Sassanid dynasty (figure 1) in Persia was established by *Ardeshir* *I*, son of *Papak* ruled one of the most influential empires in the world history and with the Romans and Byzantines were global authorities in ancient times (11, 12). Sassanid Empire was established based on strength, military, politics and science (12). There are many significant scientific achievements in this period (13). Medicine was well-organized in the official Sassanid structure. Medical centers and hospitals were built near medical universities for the first time in history. Hospitals progressed all over Persia during their empire (14). Also, the first medical symposium was held on *Khosrow* *Anoushiravan’s* (one of the greatest Sassanid kings) order in 550 CE (figure 2). 

**Figure 1:**
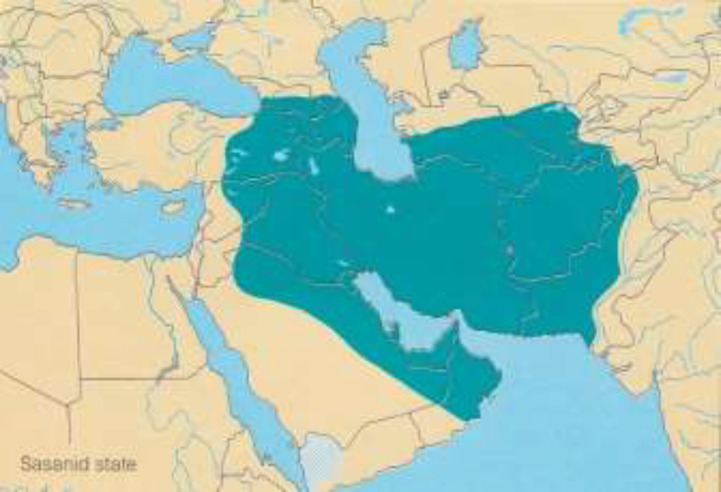
Sassanid Empire (224–637 CE)

**Figure 2:**
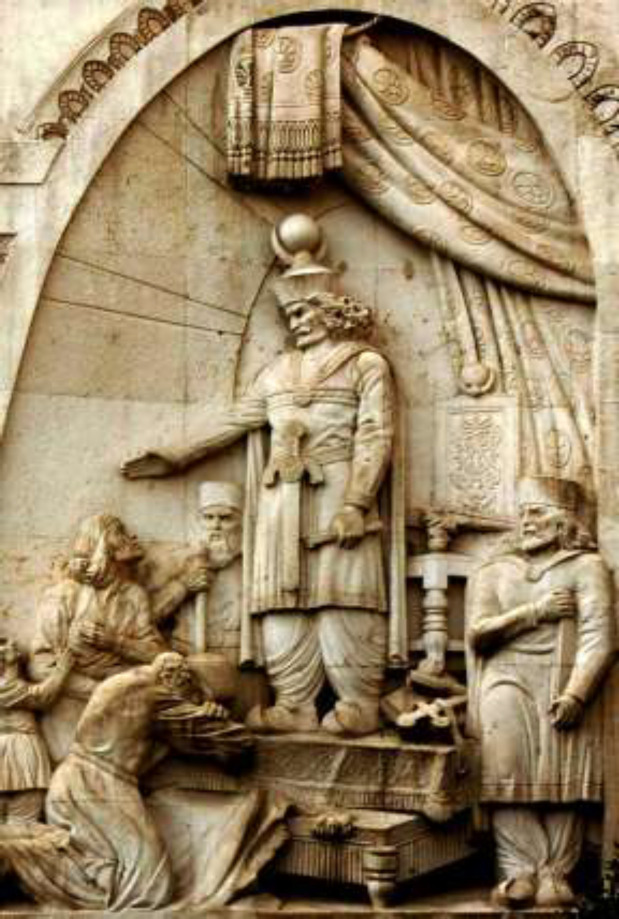
Khosrow I statue in Tehran courthouse (made by Gholamreza Rahimzadeh Arjang in 1940)

Jundishapour School of Medicine located in southeastern Iran, the most important medical center in antiquity was established during the king *Shapur* *II* reign (309 – 379 CE) (13). It was an international school attracting scholars and physicians from numerous countries like Greece, Syria, Egypt, India, as well as Persia (15). Indian, Greek, Syrian, and other cultures were considered in this era through the translation of their important books into Pahlavi or Middle Persian language (literary language of the Sasanian empire) (11). Wisdom was one of the most important issues in Sassanid science and thinking. There were many books on wisdom in that era (16). One of the most important books written during the Sassanid era, which is a handbook of wisdom and moral advice and discusses various life issues, is *Minooye Kherad*.

***Minooye Kherad*** **:** The history of didactic literature, particularly advisory literature is very long in the Persian literature. Advisory literature in its special meaning became popular in the Sassanid era. *Minooye Kherad* is a Zoroastrian manuscript (*Behdinan*) of wisdom and advice which was written in the late Sassanid era of the Middle Persian period during the 6th century CE in the Pahlavi language (17). This book probably was written during the reign of *Bozorgmehr*, the wise minister and advisor of Khosrow I (*Anoushirvan*). 

This rather brief manuscript was full of the wise and beautiful religious and practical advice in various fields (18).

It is one of the cultural monuments of ancient Persia and there are some contents about "Creation" and "Mythology" in it as well. This book has an introduction in the form of 62 ethical questions with an imaginary person named *Dana* (means wise) and the answers by *Minooye Kherad* (spirit of wisdom), presents the essence of the truth. Most of the answers are persuasive and moral-behavioral aspect. Question 15 of the book asks about the types of foods such as wheat, milk, dates and the benefits and harms of alcoholic beverage. 

**Advantages of wine consumption in** ***Minooye Kherad*** **and comparison with current medicine: **In this book, first the benefits of moderate wine drinking are explained; including improves digestion of food, increases body temperature, increases intelligence, memory, semen and blood, relieves sadness and pain, and also facial color improvement, recalls forgotten thing, improves eyesight and hearing and speech ability, performs tasks well, good thinking, improve sleep, easy to wake up and positive psychological effects (19). 

Nowadays it has been proven that patterns of moderate drinking, have been associated with a key health benefit. Relaxation, mood elevation, stress reduction and increase in sociability, are the most frequently reported psychosocial advantages of wine drinking in small amount (20, 21). Numerous recent studies have demonstrated that moderate alcohol use may have a positive effect on cognitive function such as thinking, reasoning, and remembering especially for women (22) and also, noticeably decreases risk of the incidence of dementia (23). Alcohol disrupts the balance between stimulating and inhibitory incidents in the brain, causing sedation and anxiolysis (24). Cross-sectional analysis confirmed a significant protective association between the moderate alcohol consumption (1-2 drinks/day) and age-related hearing function in older adults (aged > 50 years) compared with nondrinkers (25).

**Disadvantages of excessive wine consumption in** ***Minooye Kherad*** **and comparison with current knowledge: **In this manuscript, the physical, psychological and social harms and consequences of alcoholism including a decrease in intelligence and memory, semen and blood, damages liver and causes liver diseases, decreases muscle strength and physical ability, decreases vision, hearing, speech ability, depression and anxiety, sleep disorders, violence and aggression, causes to forget praising God, disruption of family and social relationships and negative psychological effects (19). As it is mentioned, the hepatotoxicity effect of wine is pointed in this historical text. Although there is not any more information about this effect because this text is not a medical text; it shows that the hepatotoxicity of wine as alcoholic beverage was familiar in Sassanid era. It is important historically because it is the first report in the history. 

Furthermore, other claims of the harms of wine abuse in *Minooye Kherad* could be supported by current investigations in literature. Alcoholic beverage consumption has been enlarged with industrialization of alcohol production in 1800s (26). In recent decades wine drinking have been widespread, with an average age of first use of 15 years all over the world and this remedy contributes to 100,000 deaths per year in the U.S. alone (27). Therefore, ideal medical care in current medicine is seriously affected by alcoholism, due to adversely affecting several body organs (28). 

Heavy drinking progresses neuronal loss, a variety of pathologic neurochemical pathways and a more permanent deficiency that was recognized as alcoholic dementia (29, 30). Alcohol consumption more than three standard drinks per day increases the risk for heart attack, coronary artery disease, cardiac arrhythmias, congestive heart failure and hypertension (31). Studies showed that daily, chronic and heavy alcohol consumption reduces muscle strength, also, irreversible muscle damage and enhancement in the activity of plasma creatine kinase is caused by high doses of alcohol (32).

In the stomach, heavy alcohol consumption can disturb the gastric mucosal barrier and leads to acute and chronic gastritis (33). Moreover, it causes malabsorption in the small intestine and pancreatitis (34). Chronic alcohol consumption is related to microcytic and macrocytic anemia (24). Daily alcohol consumption has a negative effect on the volume and morphology of semen but does not appear to alter its quality (35). Correlation between alcohol use and an enhancement risk of cataract (36) and beer consumption and exudative macular degeneration were reported from cross-sectional studies (37). Several studies demonstrated that the prevalence of past 30-day wine consumption and lifetime drunkenness among adolescents occur psychological distress (anxiety-induced depression) (38). Moreover, alcohol may enhance aggression among heavy drinkers (39). Alcoholic liver disease is one of the most prevalent causes of chronic liver disease worldwide. The severity of liver damage associated with alcohol varies among diverse individuals. Alcohol consumption creates a spectrum of histologic abnormalities and damage in the liver, such as steatosis (fatty liver), steatohepatitis (alcoholic hepatitis), fibrosis and cirrhosis (40). Some signs, symptoms, and abnormal findings of laboratory tests help diagnose several stages of alcohol-induced liver damage. Steatosis is frequently asymptomatic in ambulatory patients and hepatomegaly is usual in hospitalized patients. Alcoholic steatohepatitis may be asymptomatic with hepatomegaly, but splenomegaly, encephalopathy, fever and jaundice are common in hospitalized patients (41). 

Patients with cirrhosis progress evidence of hepatocellular dysfunction (e.g., jaundice and cachexia) and portal hypertension (e.g., hepatic encephalopathy, ascites and gastrointestinal bleeding) (40). Alcohol drinking causes these diseases by the inhibition of tricarboxylic acid cycle and oxidation of fat (42), reduction of phosphatidylcholine levels in hepatic mitochondria, enhancement formation of intracellular free hydroxy-ethyl radical, reducing oxidase activity and O2 consumption (43). 

## Discussion

Alcohol is the most largely used and abused drug all over the world, and its manufacturing and consumption have been mixed into mankind cultures for thousands of years. The balance between the social advantages of wine and its negative subsequences have been widely considered for centuries and many societies have struggled to limit or even remove its consumption, but alcohol drinking is inseparably connected to modern society. The historical text of *Minooye Kherad* shows the historical root of this challenge in more than 1500 years ago. It seems that this text tried to respond and find an answer for this social concern. Furthermore, current investigations approved most of the claims as benefits and harms of wine consumption in this historical text and it shows the advancement of medical knowledge about this issue in that era that we can find the perspective of this knowledge in this non-medical text. However, the most important claim in this text could be mentioned as pointing to the hepatotoxicity of wine. Although, the concept of the alcohol hepatotoxicity as one of the most important complications of alcoholism is now widely accepted, it is explained in the *Minooye Kherad*, a Zoroastrian manuscript in the late Sassanid era, dating back 1500 years ago. 

In conclusion description of the liver disease and damage caused by excessive wine consumption in *Minooye Kherad* can be considered as an early report of hepatotoxicity of wine and as a critical point in the history of misuse of alcohol.

## Conflicts of Interest:

None declared.
